# Regional Differences in End-Diastolic Volumes between 3D Echo and CMR in HLHS Patients

**DOI:** 10.3389/fped.2016.00133

**Published:** 2016-12-12

**Authors:** Alberto Gomez, Ozan Oktay, Daniel Rueckert, Graeme P. Penney, Julia A. Schnabel, John M. Simpson, Kuberan Pushparajah

**Affiliations:** ^1^Department of Biomedical Engineering, King’s College London, London, UK; ^2^Biomedical Image Analysis Group, Imperial College London, London, UK; ^3^Department of Congenital Heart Disease, Evelina London Children’s Hospital, London, UK

**Keywords:** volume estimation, ventricular function, ultrasound imaging, cardiac magnetic resonance, image registration

## Abstract

Ultrasound is commonly thought to underestimate ventricular volumes compared to magnetic resonance imaging (MRI), although the reason for this and the spatial distribution of the volume difference is not well understood. In this paper, we use landmark-based image registration to spatially align MRI and ultrasound images from patients with hypoplastic left heart syndrome and carry out a qualitative and quantitative spatial comparison of manual segmentations of the ventricular volume obtained from the respective modalities. In our experiments, we have found a trend showing volumes estimated from ultrasound to be smaller than those obtained from MRI (by approximately up to 20 ml), and that important contributors to this difference are the presence of artifacts such as shadows in the echo images and the different criteria to include or exclude image features as part of the ventricular volume.

## 1 Introduction

Accurate estimation of ventricular volumes is critical for a number of clinical applications, particularly in patients with congenital heart disease (CHD). In hypoplastic left heart syndrome (HLHS), the left heart structures are underdeveloped to the extent that they are unable to support the systemic circulation. The right ventricle (RV) is dilated and hypertrophied as a consequence, supporting the systemic circulation on its own. This results in abnormal RV geometry.

Ultrasound (US) is the most widespread cardiac imaging modality. However, image quality can be poor compared to other non-invasive techniques such as cardiac magnetic resonance (CMR) imaging. CMR is considered to provide reference images of the heart (1) and hence CMR images are frequently used to estimate reference values for cardiac shape, size, and function, as discussed by Kjaergaard et al. and Greupner et al. (2, 3). Previous studies, for example Bell et al. (4) have compared ventricular volumes obtained from CMR and echo. Moreover, numerous studies, summarized in Ref. (5), showed that echo-derived end-diastolic volumes (EDV) systematically underestimate EDV values derived from CMR images by up to 20 ml in average and up to 34% in relative terms. These differences are more significant in CHD patients than in healthy subjects or in other patient groups.

The objective of this study is to investigate the spatial distribution of the difference in reported EDV between MR-derived segmentations and echo-derived segmentations in HLHS patients. In particular, we analyze what features of the image lead to differences in contour delineation, and where these differences occur. The contribution of this paper is to describe the spatial distribution of differences between volume estimates obtained from paired CMR and echocardiographic (echo) images.

## 2 Materials and Methods

### 2.1 Patient Selection and Data Acquisition

We study multimodal images acquired from 5 patients with hypoplastic left heart syndrome (HLHS), 3 post-Norwood 1, and 2 post-Hemifontan, with an age range of 0.18–3.40 years, and weight in the range of 4.93–15.4 kg. These patients underwent a research ultrasound examination immediately after the clinical MR examination, both under general anesthesia (GA). Transthoracic ultrasound volumes were acquired using a Philips iE33 system and a cardiac X5-1 3D transducer, from subcostal windows.

Cardiac magnetic resonance (CMR) imaging was performed using a 1.5 T MRI scanner (Philips Intera Achieva, Philips Healthcare, Best, Netherlands). RV volumes and function were obtained as part of a comprehensive functional evaluation. In accordance with our unit protocol for CMR evaluation of HLHS, a single stack of contiguous 6–8 mm balanced SSFP slices (TR 1.8 ms, TE 3.5 ms, FOV 180–320, 40 phases per cardiac cycle, 6–12 lines per segment depending on heart rate, acquired resolution 1.2 mm × 1.2 mm to 1.8 mm × 1.8 mm) oriented in a plane equivalent to the short axis of the tricuspid valve were obtained in an end-expiratory breath-hold of 4–7 s per slice.

This study was carried out in accordance with the principles of the Declaration of Helsinki. Ethical approval was granted by the local ethics committee “Advanced Echocardiography in Pediatric Patients” at Guy’s and St. Thomas’s and King’s College London (09/H0802/116) after informed consent was obtained from the patients parents.

### 2.2 Ventricle Segmentation

Segmentations on both MR and ultrasound images were done manually. Semiautomatic methods based on processing of the image data, such as model based segmentation, level sets, region growing, and other methods can introduce a bias in the comparison because MR and ultrasound images perform very differently on them. Consequently, we have used the manual segmentation tool provided by the MITK software (6) to segment both modalities. The segmentation was carried out by contouring the endocardium on a stack of short-axis planes and then interpolating the contours to form a volume.

### 2.3 Ultrasound to MR Image Alignment

Echo to CMR alignment (registration) is a challenging problem. Image features are normally not consistent between the two modalities because not all structures that are visible in the ultrasound image (e.g., trabeculae, valves) are also visible in the CMR image. Moreover, view-dependent artifacts characteristic to ultrasound imaging (e.g., shadows, reverberations) lead to erroneous image features that obviously are not present in the CMR image. Last, structures that are visible in both modalities are captured in a very different way: for example, ultrasound image formation can cause thickening or narrowing of these structures depending on the angle of incidence of the ultrasound wave, while no such view dependency takes place in CMR image acquisition. As a result, most automated and semi-automated, image- or feature-based registration algorithms fail to align CMR and echo images accurately.

For this study, we have carried out image alignment by manually selecting corresponding ventricular landmarks in both modalities and calculating the rigid transform (rotation and translation) between the two landmark sets. An independent operator carried out registrations for all patients to ensure that the same alignment was used when comparing segmentations carried out by different experts.

**Figure 1:**
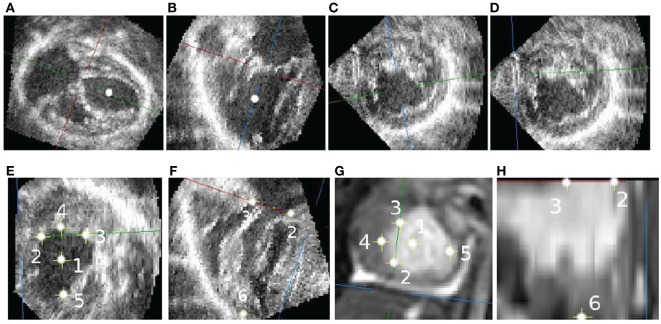
**Selecting corresponding landmarks in echo and CMR images**. **(A)** Long axis slice. **(B)** RVOT view. **(C)** Short-axis view with arbitrary rotation. **(D)** Short-axis view parallel to the diaphragm. **(E)** Valve-plane landmarks. **(F)** Valve and apex landmarks. **(G)** CMR landmarks. **(H)** CMR landmarks.

Landmark selection is done as follows: first, the base-to-apex axis was found (Figures 1A,B). Along this axis, a point at mid-height of the ventricle (represented in the figure by a white dot) is selected to produce a short-axis slice (Figure 1C). On this short view, the in-plane axes are rotated and translated so that one plane is parallel to the diaphragm, and the other passes by the closest papillary muscle (Figure 1D) while maintaining the slicing planes orthogonal to each other.

Without changing the orientations of the slicing planes, the crosshair is translated following the through short-axis direction to the atrioventricular valve plane (Figures 1E,F). At that location, six landmarks are selected: the center of the atrioventricular valve (1), the inferior (2), anterior (3), left (4) and right (5) sides of the valve annulus, and the ventricular apex (6). Following the same process, corresponding landmarks are selected in the CMR image (Figures 1G,H). The rigid transformation between the two point sets was found using the least squares method described by Arun et al. (7).

**Figure 2:**
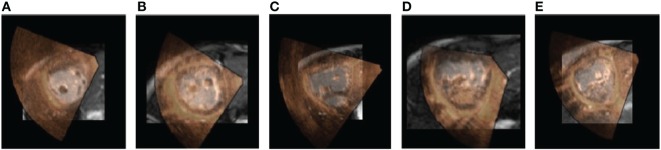
**Alignment results**. The figure shows a 2D short-axis slice of the aligned volumes for 5 patients. The CMR image is shown in the background in grayscale and the echo image is overlaid on top using a red-to-yellow colormap. A selection of movies showing the achieved alignment and its consistency over time are included in Supplementary Material. **(A)** Pat 1. **(B)** Pat 2. **(C)** Pat 3. **(D)** Pat 4. **(E)** Pat 5.

Figure 2 shows the image registration results for 5 patients. A superimposition of the medial short-axis slice from both CMR and echo images, for each patient, is shown. A selection of movies showing the achieved alignment and its consistency over time are included in Supplementary Material.

### 2.4 Regional Division of the RV

In order to carry out a regional analysis of the difference in estimated volume, we need to divide the ventricular volume into sectors. There is little literature on regional analysis of the right ventricle (RV) in Fontan patients like those with hypoplastic left heart syndrome (HLHS). For repaired Tetralogy of Fallot (ToF) patients, Zhong et al. (8) proposed a 15-segment subdivision.

This subdivision is, however, not well suited for single ventricle circulation because of the essential differences in RV morphology between the two cases. In repaired ToF, the RV morphology is not very different from a normal RV. In Fontan circulation, there is no functional left ventricle (LV), hence the RV supports the systemic circulation and has adapted its morphology becoming more globular, toward the shape of a normal LV.

For this reason, other means of describing the RV anatomy in a standardized way have been proposed. Menon et al. (9) carried out regional analysis from 2D echocardiography by dividing the myocardium in a parasternal long-axis view into four sections and in a four chamber view into 6 segments. Wong et al. (10) carried out a 3D analysis on RV morphology and function in HLHS using a population-based atlas, which defined ventricular anatomy with respect to the position of the LV remnant. This representation allowed the estimation of regional strain dividing the RV into basal, medial, and apical layers.

**Figure 3:**
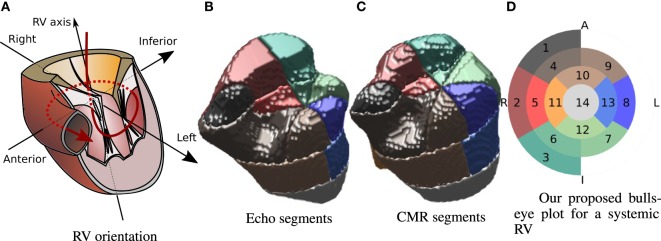
**Segment division on the RV**. Representation of the RV and its main axes **(A)**. Example of segment division for patient 2 from echo **(B)** and CMR **(C)**. Bulls-eye plot representation of the proposed 14-segment systemic RV division **(D)**. Annotations indicate left (L), right (R), inferior (I), and anterior (A).

Inspired by the divisions carried out by Menon et al., Zhong et al., and Wong et al. (8–10), and taking into account the standard AHA 17 segment division for the LV proposed in Ref. (11), in this paper, we propose to divide the 3D RV shape into 14 segments as indicated in Figure 3. Along the RV axis, four layers are defined: apical, medial, basal, and valvular. The apical layer consists of a single sector. The medial layer and the basal layer are divided into 4 and 6 sectors, respectively (similar to the LV AHA division). The valvular layer is divided into 3 sectors that cover half of the circle reflecting the asymmetric shape of the RV, and cover the “shoulder” area underneath the inflow valve. This layer covers the upper anterior wall (1), the upper lateral free wall (2), and upper inferior free wall (3). The basal layer, situated below the valvular layer, starts at the intersection point with the anterior (4), lateral (5), and inferior (6) parts of the free wall and the inferoseptal (7), lateral-septal (8), and anteroseptal (9) sectors. The medial layer includes four sectors covering the anterior (10), the lateral free wall (11), the inferior (12), and the septal (13) sectors. The apical layer includes a single sector (14). Figure 3A shows a representation of a RV for reference. Figure 3 shows a 3D representation of the segment division for one patient from the echo-derived segmentation and from the CMR-derived segmentation. Colors are matched by the bulls-eye plot diagram in Figure 3D, which is used as model for the results in the remainder of this paper. The orientation of the bulls-eye plot and the denominations “anterior” (A) and “inferior” (I) are consistent with that in Ref. (11). We have replaced the septal and lateral names in (11) by right (R) and left (L), because we believe this is a more straightforward and intuitive notation in systemic RV patients.

An advantage of the landmark selection process described in Sec 2.3 is that it allows to define the segment division automatically since the RV axis is defined by points 1 (center of tricuspid valve) and 6 (RV apex), and the superior-to-inferior direction is defined by the points 2 and 3.

### 2.5 Experiments and Data Analysis

Three experts carried out EDV segmentations on 5 pairs of pre-aligned MR and ultrasound images, as described in Sec. 2.2. These images were spatially aligned as indicated in Sec. 2.3. The resulting aligned segmentations were divided into 14 segments as described in Sec. 2.4.

The resulting end diastolic volumes (EDVs) are compared globally (*ΔEDV = EDV_echo_ − EDV_CMR_*) in absolute terms, and also regionally. For the regional analysis, the difference between echo-derived and CMR derived regional volumes is expressed as a fraction of the total EDV volume estimated from CMR:

(1) $${\mathrm{\Delta EDV}}_{r}=\frac{{\mathrm{EDV}}_{r,\mathrm{echo}}-{\mathrm{EDV}}_{r,\mathrm{CMR}}}{{\mathrm{EDV}}_{\mathrm{CMR}}},\text{for every region }r$$

This allows us to compare the obtained values across patients. In order to compute statistics on segmentations from multiple experts, the average segmentation was computed by averaging the binary masks representing the EDV segmentations followed by a thresholding operation where voxels with an intensity greater than 0.5 were kept.

In addition to the numerical analysis, we have carried out a qualitative analysis by comparing the average segmentation contours at four different short-axis planes uniformly spaced along the RV axis for each patient.

## 3 Results

### 3.1 Numerical Results

Global EDV differences are shown in Table 1. The numbers reflect the average ± SD over all experts, for each patient and each modality, in milliliters. The numbers reported are within the normal range and variability to other studies in the literature as reported by Simpson et al. (5).

**Table 1:** **Ventricular volumes at end diastole in milliliters, including segmentations from all experts**.

Patient	Echo	CMR	*EDV_echo_−EDV_cmr_*
1	29.69 ± 2.67	57.73 ± 16.98	−28.05 ± 14.75
2	18.27 ± 1.27	27.70 ± 5.58	−9.44 ± 4.37
3	31.17 ± 2.54	51.17 ± 8.59	−20.00 ± 9.61
4	26.23 ± 5.90	24.95 ± 10.12	1.29 ± 7.74
5	21.17 ± 3.28	26.97 ± 9.87	−5.79 ± 6.96

Large variability in the CMR derived volumes in patient 1 are associated to low echo image quality (as shown in Figure 6, top left), which led to large differences on how experts decided to include some structures like the papillary muscles.

**Figure 4:**
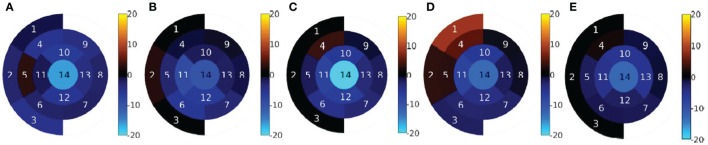
**Regional volume difference relative to the global CMR-derived volume, in percentage**. **(A)** Pat 1. **(B)** Pat 2. **(C)** Pat 3. **(D)** Pat 4. **(E)** Pat 5.

**Figure 5:**
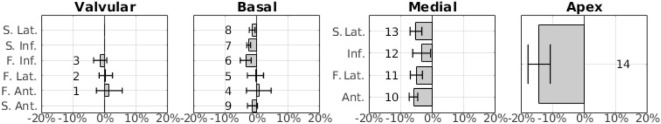
**Average regional volume difference relative to the global (CMR)-derived volume between the regional CMR derived volume and the echo-derived volume, in percentage**. The error bars indicate ± SD.

The results of the regional analysis of EDV differences are shown in Figures 4 and 5. Figure 4 shows the average relative difference in regional EDV bulls-eye plot for each patient, in percentage. There is a common pattern across all datasets where the highest disagreement is near the apex, decreasing gradually near the valve plane.

Figure 5 shows the integrated results from all patients as a bar chart. This representation has been chosen instead of the bulls-eye plot in order to accurately show the average values as well as the SD as error bars. The sector number corresponding to each bar is indicated next to the bar. Note that, in the basal layer, the order of the bars has been modified so that sectors that are approximately aligned vertically are represented as bars that are aligned horizontally.

The results shown in Figure 5 are consistent with the results shown in Figure 4E. The highest average disagreement takes place at the apex, with a −14.2% relative difference between echo-derived volume and CMR-derived volume. The spatial distribution of the error in the medial layer is uniformly distributed and close to −5% in average. In the basal and the valvular layers, the difference between the two modalities is significantly smaller. Interestingly, in sectors near the outflow tracts (1, 2, 4, and 5), there is a high variance across patients and operators. The qualitative results in the next section expand on the potential reasons for this.

### 3.2 Qualitative Results

**Figure 6:**
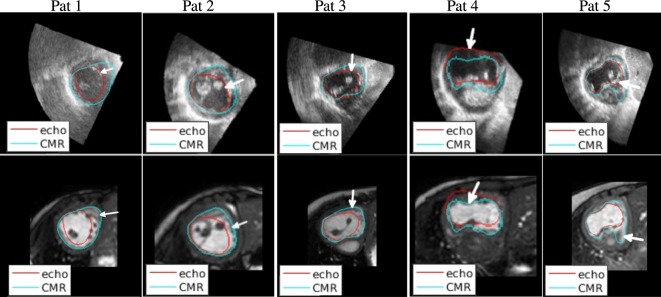
**Qualitative results**. Visual comparison of the echo-derived segmentation and the CMR-derived segmentation superimposed on the aligned echo image (top row) and on the aligned CMR image (bottom row).

Figure 6 shows a selection of short-axis, end-diastole views of both CMR and echo including the outline of both echo and CMR-derived segmentations (after alignment), to illustrate the most significant findings of this study. The ventricular segmentation from echo is represented as a red contour and the CMR-derived segmentation as a green contour. A comprehensive collection of short-axis views for all patients at different planes is included in Supplementary Material.

The 2D slices in Figure 6 illustrate sources of systematic differences between EDV segmentations carried out using CMR images and echo images. On the first column on the left, a very significant difference between the two contours can be observed. Echo-derived segmentation was particularly challenging in this patient due to the low-image quality (compared to images from other patients). Additionally, the echo-derived contour was drawn excluding trabeculae from the segmentation. In the CMR image, where the visibility of the endocardium is poor, the segmentation appears to run closer to the myocardium.

On the second column from the left, it can be seen that image quality is relatively high in both modalities, but the difference comes from the contours delineating different structures. The echo-derived contour (in red) correctly follows an image edge (top row), and the CMR-derived contour (in blue) also follows an edge in the CMR image (bottom row). The edge in the echo image represents the trabeculations, which are not visible in the CMR image, where the contour follows the myocardium. A similar effect can be observed in the third column (patient 3).

The fourth column shows the effect of lack of boundary definition in the ultrasound image on the resulting segmentation. In this case, overall image quality is high, but the anterior wall is not visible due to shadowing from the air in the lung. As a result, the delineated contour does not match the real structure, which is visible in the CMR image. This finding is reflected in our proposed bulls-eye plot in Figure 4D.

The fifth column illustrates disagreement due to lack of boundary definition. An unclear boundary was delineated as a true boundary on the echo image, while it was considered part of the ventricular cavity in the CMR.

## 4 Discussion

In this paper, we have investigated the spatial distribution of RV volume differences by comparing ventricular segmentations from CMR and from echo after aligning the two modalities on patients with HLHS. We have found a similar level of overall volume difference (up to a 20 ml) between echo and CMR as in related literature, summarized in (5). We have found that there are two major causes for this volume difference. First, the lack of boundary definition in some echo images as a consequence of shadowing artifacts produces large errors in the segmentation. These kind of artifacts occur more commonly near the anterior-free ventricular wall because of the proximity to the lungs.

The second finding, perhaps more interesting, is that the trabeculations in the right ventricular surface are captured in a very different way in the CMR images and in the echo images. Mostly, CMR images show the inner RV surface as flat and free from the characteristic foldings and complex structures that are visible in the echo images. In these cases, the CMR segmentation lies closer to the epicardium. This partly explains the consistent bias for echocardiography to produce lower volumes than MRI.

A third, less significant cause of volume disagreement appears to be the lack of agreement between experts on where to finish the ventricular segmentation near the inlet and outlet of the RV.

In the case where echo image quality is significantly low, for example in patient 1 (Figure 6, top left), the segmentation process can be very challenging and the difference with CMR-derived volumes can be extremely large. A larger study is required to ascertain whether patient 1 is representative, in terms of image quality, of this patient group.

A limitation of the manual registration is that it can introduce a bias due to operator dependency inherent to a manual process. This would not affect overall segmentations (since segmentations are carried out before registration). We believe the impact of this potential error is relatively small since manually picked landmarks are commonly used as reference (12, 13) when a ground truth registration is not available. An interesting consideration of the proposed landmark-based registration method is that although excellent image alignment can be achieved, the landmark set alignment can yield a relatively large residual error (up to 4 mm). The reason for this residual error is that landmarks do not necessarily provide a very good pairwise correspondence, but still provide an accurate groupwise correspondence. For example, points (2–5) are picked at the intersection of specific axes with the visible valve annulus contour, but this contour can be captured differently in CMR and echo, which is consistent with the endocardial segmentations done on CMRI and echo images that we have shown.

The limited number of patients and the lack of a ground truth volume measurement prevents us from making a strong statement on which volume estimate is more accurate; however, our data suggest that, when high-quality echo data are available, RV estimates can be as good as those from CMR. This can be of particular clinical significance if not only ED volumes are required but also time-resolved volume estimations are sought, because echocardiography is uniquely placed to provide high temporal and spatial resolution images of the heart.

## Author Contributions

All the co-authors have had substantial scientific input and have agreed upon the current content.

## Conflict of Interest Statement

The authors declare that the research was conducted in the absence of any commercial or financial relationships that could be construed as a potential conflict of interest.

## Supplementary Material

Video S1 **Fused visualization of 3D echo and stack-cine CMR from patient 1**.

Video S2 **Fused visualization of 3D echo and stack-cine CMR from patient 2**.

Video S3 **Fused visualization of 3D echo and stack-cine CMR from patient 3**.

Video S4 **Fused visualization of 3D echo and stack-cine CMR from patient 4**.
