# Supplementary material for: Regional Differences in End-Diastolic Volumes between 3D Echo and CMR in HLHS Patients
Source: Front Pediatr. 2016 Dec 12;4:133. doi: 10.3389/fped.2016.00133 (PMC5152531; doi:10.3389/fped.2016.00133)
Supplement: Supplementary file 5 [file Presentation_1.PDF]

Supplementary Material: Local Differences in End Diastolic Volumes between 3D Echo and CMR in Fontan Patients

Alberto Gomez [1,*], Ozan Oktay [2], Daniel Rueckert [2], Graeme Penney [1], Julia Schnabel [1], John Simpson [1,3], and Kuberan Pushparajah [3]
*Correspondence:
Dr. Alberto Gomez
alberto.gomez@kcl.ac.uk

1 SUPPLEMENTARY FIGURES

1.1 Figures

In this section we show of short-axis views of both CMR and echo including the outline of both echo and CMR derived segmentations. Four uniformly spaced slices from each patient are included, both on the CMR background and on the echo background for easier comparison.

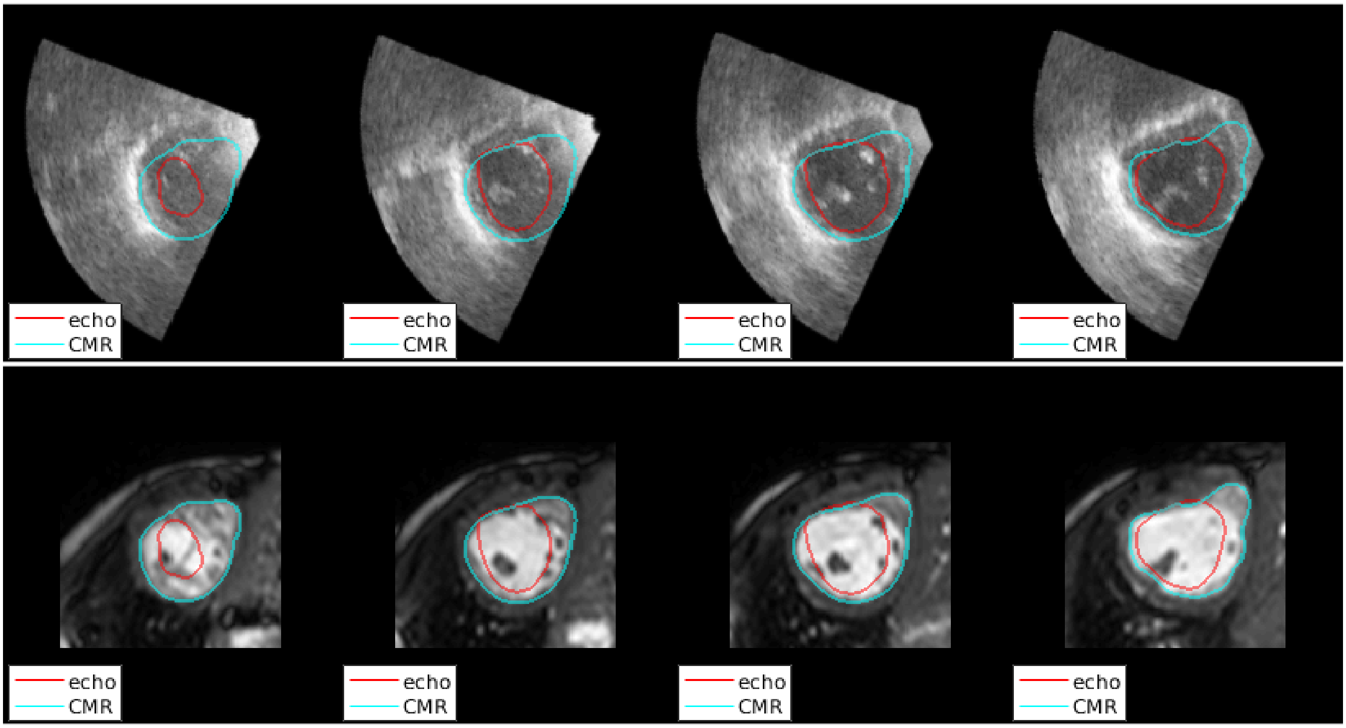

Figure S1. Patient 1

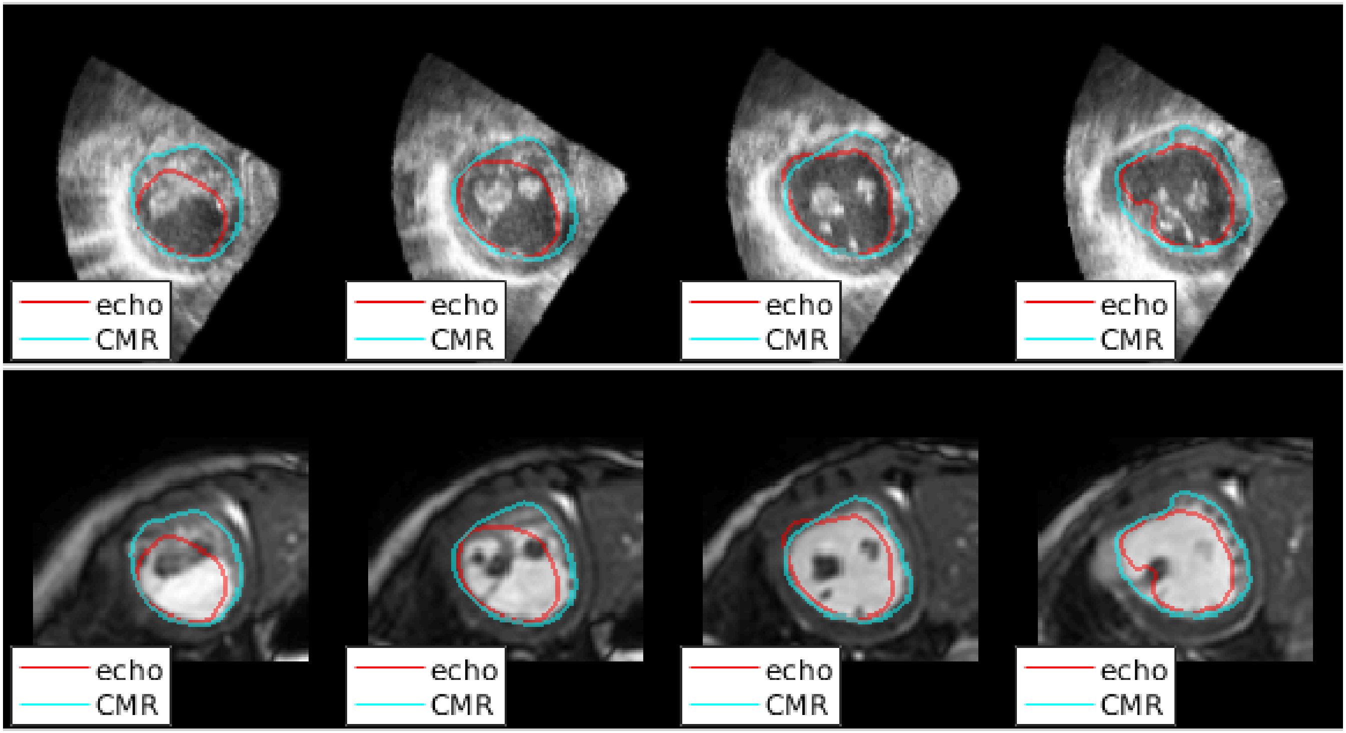

Figure S2. Patient 2

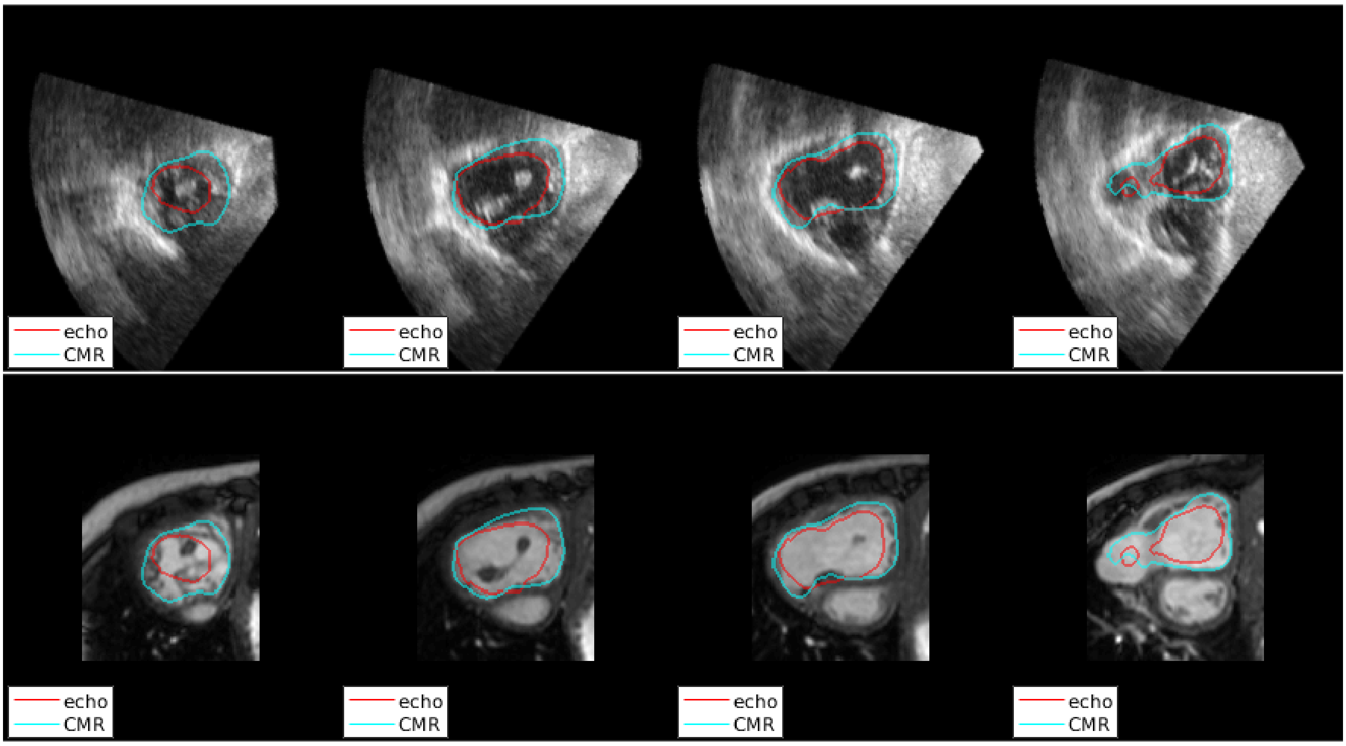

Figure S3. Patient 3

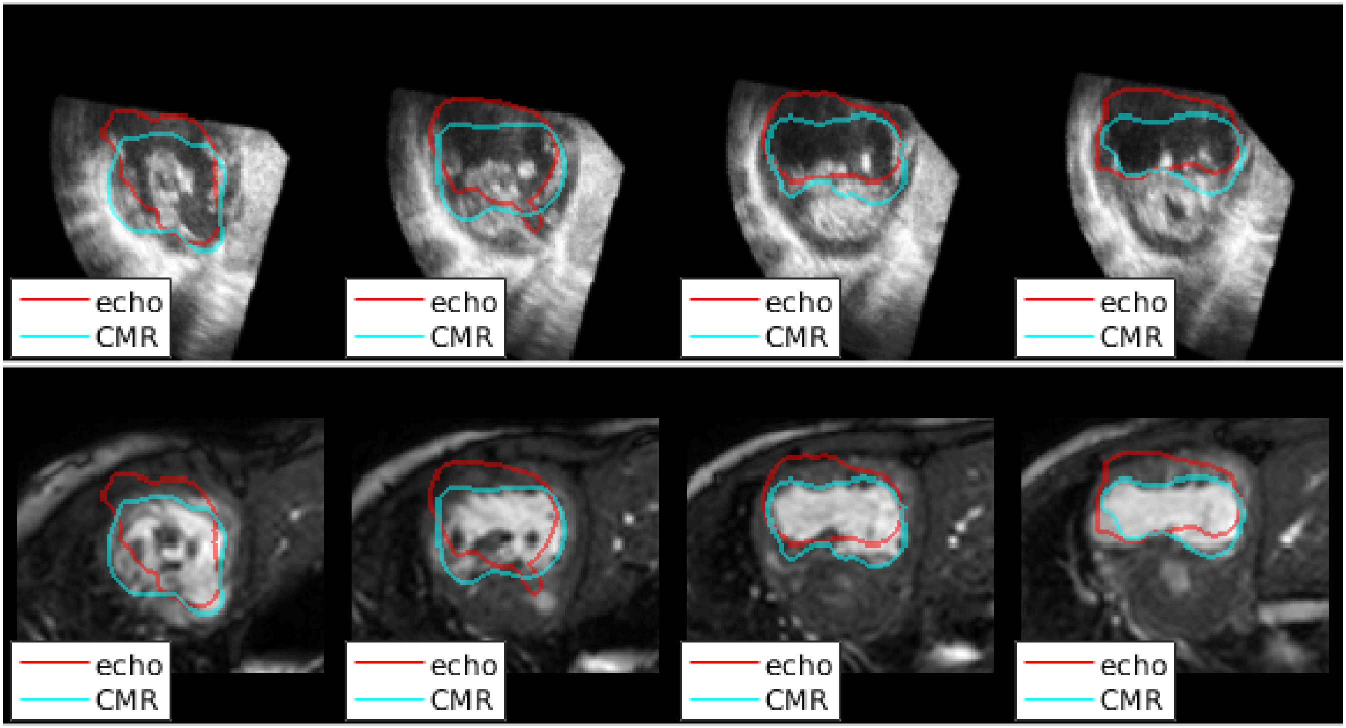

Figure S4. Patient 4

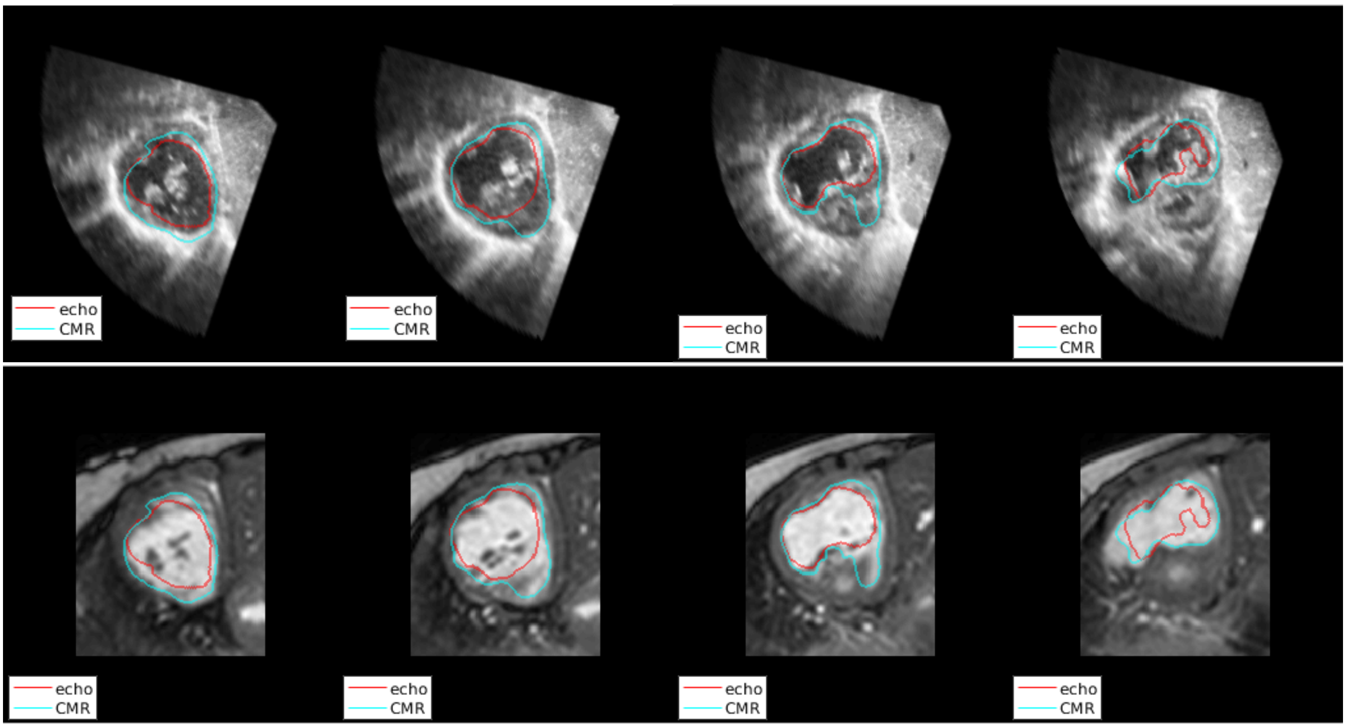

Figure S5. Patient 5
